# Cost-effectiveness of Multisystemic Therapy for adolescents with antisocial behaviour: study protocol of a randomized controlled trial

**DOI:** 10.1186/1471-2458-13-369

**Published:** 2013-04-19

**Authors:** Danielle EMC Jansen, Karin M Vermeulen, Annemieke H Schuurman-Luinge, Erik J Knorth, Erik Buskens, Sijmen A Reijneveld

**Affiliations:** 1 Department of Health Sciences, University Medical Center Groningen, University of Groningen, Groningen, PO Box 196, 9700 AD, the Netherlands; 2 Department of Sociology and Interuniversity Center for Social Science Theory and Methodology (ICS), University of Groningen, Grote Rozenstraat 31,9712 TG Groningen, the Netherlands; 3 Medical Technology Assessment, University Medical Center Groningen, University of Groningen, PO Box 30.001, 9700 RB Groningen, the Netherlands; 4 Department of Special Needs Education and Youth Care, University of Groningen, Grote Rozenstraat 38, 9712 TJ Groningen, the Netherlands

## Abstract

**Background:**

Multisystemic Therapy (MST) is an intensive, short, family- and community-based treatment for serious antisocial behaviour and delinquency in youth. It is an emerging intervention for serious juvenile delinquents. However, conclusive evidence on the balance between costs and effects is limited and in fact non-existent for the Netherlands. The aim of this protocol is to describe the design of a study to evaluate the cost-effectiveness of MST as compared to Care-As-Usual (CAU).

**Methods:**

The cost-effectiveness of MST will be assessed through a Randomised Controlled Trial. Primary outcomes aggressive and delinquent behaviour will be assessed with the parent-reported CBCL and adolescent-reported YSR. Health care utilisation, production loss, and quality of life are recorded using the self-report 'Trimbos and iMTA questionnaire on Costs associated with Psychiatric illness' (TiC-P), and with the MOS Short-Form General Health Survey (SF-20) and EuroQol -5D (EQ-5D), respectively. The study aims to enrol 100 clients in both conditions (MST and CAU). Data will be obtained before treatment (T1), immediately after treatment (T2; 5 months after T1) and at follow up (T3; 6 months after the end of the treatment) from a variety of sources, i.e. clients, parents/primary carers, professionals and police records.

**Discussion:**

Studying the cost-effectiveness of this treatment for youth antisocial behaviour is important in order to provide information to policy makers on whether the provision of this intervention represents good value for money. Introducing a cost-effective evidence based programme may result in valuable health gains for moderate costs.

**Trial registration:**

NTR1390

## Background

The direct costs associated with antisocial and delinquent behaviour of adolescents are high. Estimated expenses of incarceration amounted to 114.000 - 250.000 euro per youth per year [1,2]. The degree of recidivism among juveniles who were incarcerated has been estimated to be at least 75% in the first three years after release [3]. Besides direct costs of locking up offenders, incarceration also has wider costs and consequences. Indirect costs include amongst other things extra support for the adolescent to reintegrate into the education system or the labour market and lost wages of parents due to the high time demands of an incarcerated child [1].

As a result of the unfavourable outcomes of incarceration, there is a strong need for evidence-based treatments that can provide an alternative to incarceration for youth with serious emotional and behavioural problems. Multisystemic Therapy (MST) has been proposed as a promising, cost-effective intervention for this group of young people. Several studies show significant reductions in number of serious crimes and days incarcerated [4-6]. However, research conducted in Canada and Sweden did not show significant differences in treatment effects between MST and care-as-usual (CAU) [7,8]. A review by Littell et al. [9] confirmed that the evidence regarding the superiority of MST compared to CAU is still inconclusive.

The introduction of an intensive family- and community-based treatment such as MST will always be associated with additional costs. Cost-effectiveness studies in child and youth care are required to provide decision makers with information on whether this intervention represents good value for money. Therefore, studies are needed that assess how well MST works – its influence on behaviour and the use of health and welfare services – and how much it costs. Cost-effectiveness analysis (CEA) is an approach that combines these requirements.

Cost-effectiveness has hardly been studied in child and youth care. MST is an example of a youth care intervention. Available studies on cost-effectiveness of MST for adolescents with antisocial behaviour revealed generally positive results, but direct translation of previous findings is not possible due to several factors. For example, the study of Klietz et al. (2010) did not include the full spectrum of youth care utilization in their cost-effectiveness analysis [10]. In addition, the study of Olsson (2009) focused only on the direct costs associated with serious behavioural problems and overlooked indirect costs such as productivity loss [11]. All these methodological differences across studies lead to an incomplete overview of costs of MST [11]. To illustrate: in the study of Olsson (2009), MST was found to cost more than double that estimated in the studies of Aos et al. (2004) and Schoenwald et al. (1996) [11,12].

The aim of the present study is to assess the balance between direct and indirect costs and effects of MST in the Netherlands as compared to Care-As-Usual for adolescents with serious behavioural problems.

## Methods

### Trial design

The design of this cost-effectiveness study will be described following the CONSORT statement [13]. The study is a supplement to an MST-effectiveness study [14], which concerns a randomized controlled trial (RCT) with a follow-up measurement after 6 months. Participants will be randomly assigned to one of the two groups: MST or Care-As-Usual (CAU). Data will be collected using a standardized self-report questionnaire in a face-to-face situation before treatment (T1), immediately after treatment (T2) and at follow up (T3; 6 months after treatment) among adolescents, parents/primary carers and therapists. Adolescents and their parents will both receive €10 for filling out the questionnaire. The Medical Ethics Committee of Utrecht University approved the study design, protocols, procedures and informed consent. Participation is voluntary and all participants are asked to provide written informed consent at T1.

### Recruitment

Participants will be recruited via three MST institutions spread over the Netherlands. The recruitment per organization will differ because of differences in the setting in which MST will be delivered. One MST organization is located in the North of the Netherlands; the other two locations are in the West of the Netherlands.

In *the North of the Netherlands*, the study population will consist of adolescents referred by family guardians of the Youth Care Office to the youth care program called “Do What Works”. This program consists of maximum six weeks secure residential care, followed by MST (Intervention) or FFT (Functional Family Therapy; Care-As-Usual). After the residential period, adolescents will be randomized to MST or to Care-As-Usual. At the same time, adolescents and their parents will be informed about the MST-effectiveness study and they will be asked for their informed consent. Upon mutual consent of the adolescent and the parents, a first appointment will be made before the start of MST or Care-As-Usual, in order to obtain written informed consent and to administer the first questionnaire.

The recruitment in *the Western part of the Netherlands* will be conducted via referring agencies such as Child Protection Council, juvenile judges, Bureaus Youth Care and local referral institutions. The referrers will inform the potential eligible juveniles and their families that a study will be conducted to investigate the effectiveness of youth care. If the families meet the inclusion criteria for MST according to the MST supervisors of the participating institutions, research procedures will be explained to the juveniles and their families, and their informed consent to participate in the study will be obtained by researchers.

### Participants

Participants eligible for this study are adolescents (and their families) between 12 and 18 years old who show serious, violent, and chronic antisocial behaviour. Exclusion criteria are an IQ below 70, acute psychiatric problems that place adolescents and their family at risk for out-of-home placement, and dominant sexual problems. Adolescents may be court-ordered or referred by primary health care, social workers or self-referred.

### Interventions

**MST**. Multisystemic Therapy (MST) is an intensive, short-term, home- and community- based intervention program for families of youth with severe psychosocial and behavioural problems. It is based on the social ecological theory of Bronfenbrenner [15] and on models of the cause of delinquency [16]. MST is designed to address complex psychosocial problems and provides an alternative treatment for out-of-home placement of children and youth. It uses the various systems in which the adolescent is embedded: family, peer group, school and neighbourhood. In consultation with the members of the family, the therapist identifies a set of treatment goals and assigns the tasks required to accomplish these goals. MST lasts 4 to 6 months and takes the individual needs of the client into account. Therapists are available 7 days a week, 24 hours a day. Each team includes 3–5 therapists who have a caseload of 3–5 clients each.

**CAU**. Care-As-Usual consists of treatments that are already available for adolescents with severe psychosocial and behavioural problems: juvenile justice services, child welfare services, individual adolescent counselling and home-based social services (for instance, parental counselling). Also included and variably implemented across regions are recently developed, evidence-based services such as Functional Family Therapy (FFT) [17] and Multi-Dimensional Family Therapy (MDFT) [18].

### Randomization

Immediately after referral (Western part of the Netherlands) or after the residential period (Northern part of the Netherlands), participants will be randomized to MST or CAU with the use of a computerized randomization program. This program will execute the randomization separately for each site. The ratio of randomization between MST and CAU will be 1:1.

### Outcome measures

In cost-effectiveness studies, costs are linked to treatment or clinical outcomes. All outcomes will be assessed at all three measurement moments.

#### Primary outcomes

Primary outcomes of this study include: recidivism and the frequency and seriousness of antisocial behaviour and other types of problems (internalizing problems, substance use). *Recidivism* will be assessed with data of the Research and Policy Database for Judicial Documentation. With this data we will assess the number of arrests, type and severity of offence of adolescents re-offending during the research period.

*Prevalence and seriousness of antisocial behaviour* will be assessed via different informants and instruments. First, parents and adolescents are asked to fill in the 33 items of the Delinquent Behaviour scale and the Aggressive Behaviour scale of the Child Behaviour Checklist (CBCL: 33 items) [19] and the Youth Self Report (YSR: 30 items) [20,21] respectively. For each item, the child’s behaviour has to be rated on a three-point scale, ranging from 0 (never) to 2 (often).

Additionally, adolescents will fill in two subscales of the Self-Report Delinquency scale (SRD) were used to assess self-report delinquency [22]. Adolescents will be asked to indicate on a list of potential delinquent behaviours whether they engaged in the described behaviours during the past 6 months (‘yes’ or ‘no’). The SRD ‘Violent offending’ (5 items) and ‘Property offences’ (10 items) scales will be used.

*Other types of problems* of the adolescent firstly comprise anxiety/depression, withdrawal and somatic complaints. These items will be measured with 25 items of the Youth Self Report [20,21].

In addition substance use (use of alcohol, soft and hard drugs) will be measured with several questions from a Dutch questionnaire used in the study Youth and risky behaviour [23].

#### Costs items

In addition to change in primary outcomes, the costs of Multisystemic Therapy compared to CAU will be assessed. Cost assessment will be done from a societal perspective, over a time horizon of one year.

Cost components of adolescent antisocial behaviour will be measured by a modified version of the Trimbos/iMTA questionnaire for Costs associated with Psychiatric Illness (TiC-P) [24]. This questionnaire is originally designed to measure direct and indirect costs of mental health problems. For the current study, we adjusted the questionnaire to a version more suitable for assessments of costs related to child antisocial behaviour [25]. We did this mainly by adding items about youth and social care. Direct costs of the interventions as well as indirect costs will be assessed via various informants, namely professionals, parents and adolescents.

*Direct costs* concern use of all resources that is directly related to the intervention and will be estimated through the review of (time) investment of the professionals, including training, overhead, communication with relevant others, and time and travel costs of professionals involved. In addition, direct costs of health and youth care utilization will be assessed via parents (among other things: visits of the adolescent and parent to health and youth care professionals, hospitalizations and residential treatments, contact with judicial authorities and medication use).

*Indirect costs* are characterized as productivity losses, due to time lost from work because of the antisocial behaviour of the adolescent [26]. Data on productivity loss will be collected via the Short Form Health and Labour Questionnaire (SF-HLQ) that is part of the TiC-P [24]. The Short-Form HLQ (SF-HLQ) contains questions about absence from work, reduced efficiency at work and difficulties with job performance.

#### Unit prices and cost calculations

Estimates of unit costs will be based on the Dutch guideline prices [25]. Costs of medication use will be based on the listed prices, including value added tax, and a pharmacists allowance. Costs due to productivity losses will be based on the overall mean hour productivity cost for men and women, calculated according to the human capital approach [27]. Multiplying the respective volumes of resource use with their corresponding unit prices will result in the associated total costs. Costs will be calculated in the European currency (Euro). The price level used will be of 2011.

*Quality of life* will be assessed with the EuroQol (EQ-5D) and the Short Form-20 (SF-20), which are validated tools for measuring general health-related quality of life [28,29]. The EQ-5D is a self-administered, generic, health-related quality-of-life questionnaire that contains two sections, a descriptive section and a valuation section. The *descriptive* section consists of five dimensions: mobility, self-care, usual activities, pain/discomfort and anxiety/depression. Each dimension has three levels of severity: no problems, some/moderate problems, and extreme problems. For each severity level a valuation is available that is based on the judgment of the general public about the value of this health state. Values on the five dimensions will be transformed into one single score by applying a formula, ranging from 0 to 1 (with lower scores representing more problems), and by using the British tariff [30]. We will use the British tariff because this is used by default in Europe, and thus the results are more generalizable and the comparison between studies is more adequate.

The *valuation* section of the EQ-5D contains a visual analogue scale, on which the participant rates his/her health on a vertical, visual analogue scale where the endpoints are labelled ‘Best imaginable health state (score of 100)’ and ‘Worst imaginable health state (score of 0)’. Valuations of the VAS (original values 0–100) will be linearly transformed into a 0 to 1 score. This valuation of health status is a so-called ‘utility’, a relative valuation of a health condition compared with perfect health and dead. This method has been found to be efficient and easy to use, and appears to provide meaningful values for relative preferences of health and treatment [31]. Thus far the EQ5D has not been validated in the domain of behavioural problems, i.e. there are no formal utilities available for severe antisocial behaviour. In a separate study we have generated valuations for serious behaviour problems in children and adolescents (Vermeulen et al., submitted).

The *SF-20* assesses six important health concepts: physical functioning, role functioning, social functioning, mental health, current health perceptions, and pain. Each dimension has five levels. For each subscale, a scale score will be calculated. The scale scores can be seen as multidimensional, health-related quality of life indicator. The internal consistency of the subscales of the Dutch SF-20 has proved sufficiently high: Cronbach's alpha for each subscale was at least .80 [32,33].

### Data collection procedure

Data will be collected using a standardized self-report questionnaire in a face-to-face situation. The questionnaires will be administered before the intervention (T1), directly after the intervention (T2) and six months after the intervention (T3). Adolescents and their parents will both receive €10 for filling out the questionnaire. The therapist will fill out a questionnaire after the treatment has finished (T2). The questionnaires are also available in old Berber for the Berber Moroccan families. In addition, we make use of one Arab translator who will visit all Arab families.

### Sample size

Sample size will be based on testing differences in outcomes between MST and CAU, at .80 power, an alpha of .05 and a medium effect size [34]. This leads to 64 participants per group. It should be noted that the power to detect a difference in costs and/or other outcomes is extremely difficult to assess a priori. Since this is a first attempt to perform a cost-effectiveness study in this area and no pilot data are available (allowing an assessment of variability) we cannot present a formal power calculation. However, to determine a clinically relevant effect size of 0.5 with a power of 80% at an alpha of 0.05, we will need 81 in each treatment arm, making 2 × 100 a probably safe sample size [35].

### Statistical analysis

First, descriptive statistics will be performed to describe the demographic characteristics of the study population.

Second, primary measures of effect will be analysed using within group comparisons (pre-post treatment) and between group comparisons. The latter one will use difference scores (pre-post intervention) after which the treatment effects of the two treatment conditions will be compared. Statistical testing will be done using either the paired t-test or the Wilcoxon signed-rank test for comparisons within the treatment groups and the unpaired t-test or the Mann–Whitney U test for comparisons between the treatment groups.

Third we will describe the mean costs per respondent in both treatment groups. Differences in costs between the intervention group and the control group will presented based on the trial data, and 95% confidence intervals will computed based on bootstrap re-sampling with 5000 replications of the trial data. Separate estimates will be made for different cost categories. For the cost-effectiveness analysis, mean annual societal costs will be linked to the primary measure of effect (Aggressive behaviour and Delinquent behaviour). A cost utility analysis will link the EQ-5d based utilities to the cost.

Fourth, point estimates for the incremental cost-effectiveness and cost utility ratio (ICER) will be computed on complete cost-effect pairs by dividing the incremental societal costs by the incremental effects at 12 months. Uncertainty around the ICERs will be estimated using bootstrapping, generating 5000 replications of the original dataset. A cost-effectiveness acceptability curve (CEAC) will be generated to represent the probability that MST is cost-effective compared to usual care over a range of thresholds [26,36].

## Discussion

This paper presents the design of a randomized controlled trial to investigate the cost-effectiveness of MST in the Netherlands, an intervention program for families and youth with severe psychosocial and behavioural problems.

Studying the cost-effectiveness of this intervention is important in order to provide information to policy makers on whether the provision of this intervention represents good value for money. Introducing a cost-effective evidence based program will result in substantial health gains at acceptable costs. MST can be easily embedded in both usual mental health care and welfare services in The Netherlands.

### Strengths and limitations

This study will contribute to the extension of scientific knowledge about economic evaluation in the field of child and youth care. This concerns the evaluation of the actual balance between costs and effects in a field of care where this has hardly ever been explored before, i.e. with very limited sources to derive costs of other outcome valuations from. The study will be conducted as part of a randomized controlled trial. By using such a design, it can be assumed that any confounding variables are cancelled out.

Furthermore, the study will be conducted as a multicentre trial. This will increase the generalizability of the study because this will lead to a large number of participants, different geographic locations and the ability to compare results among organizations. We will obtain both direct and indirect costs across the entire field of youth care. We will also include quality of life measures. To increase inclusion and prevent loss to follow up, all adolescents and parents will receive assistance in administering the questionnaires. Finally, most outcome variables will be assessed via two informants. Multi-informant assessment is recommended as a way to obtain a more complete picture of the adolescent than reliance on self- or parent-report alone [16].

Despite the strengths and innovative aspects of this cost-effectiveness study, there are some issues that our study is unable to take into account. First, follow-up is limited to a period of six months. To determine long-term effects of the intervention, there should be follow-up after a couple of years to assess the effects and costs when the adolescent is older. Second, it is to be expected that we will be dealing with a ‘difficult-to-reach”-population.

## Conclusions

Cost-effectiveness is a research topic that has hardly been looked at in the child and youth care field, both internationally and nationally [37-40]. More knowledge about cost-effectiveness in child and youth care will aid policy makers, health care authorities as well as judicial authorities, to set priorities and decide on implementation of treatment modalities for youth with serious behavioural disturbances. This study can provide information on both the feasibility of CEA in this field in general, and on the cost-effectiveness of MST specifically.

## Competing interests

The authors declare that they have no competing interests.

## Authors’ contributions

SAR, EJK and EB had the original idea for the project, wrote the study proposal, and obtained funding for the study. SAR and DJ participated in the coordination of the study. AS-L and DJ conducted the study. DJ wrote the final manuscript, in close cooperation with KV. The manuscript was discussed, edited and revised by all authors. All authors read and approved the final manuscript.

## Pre-publication history

The pre-publication history for this paper can be accessed here:

http://www.biomedcentral.com/1471-2458/13/369/prepub
